# Treatment and Outcome of Metastatic Renal Cell Carcinoma With Sarcomatoid Differentiation: A Single-Center, Real-World Analysis of Retrospective Data

**DOI:** 10.3389/fsurg.2021.763271

**Published:** 2021-11-18

**Authors:** Florian Janisch, Christina Kienapfel, Constantin Fühner, Thomas Klotzbücher, Phillip Marks, Tobias Hillemacher, Christian P. Meyer, Takehiro Iwata, Mehdi Kardoust Parizi, Guido Sauter, Margit Fisch, Shahrokh F. Shariat, Roland Dahlem, Michael Rink

**Affiliations:** ^1^Department of Urology, Medical University of Hamburg, Hamburg, Germany; ^2^Department of Urology, Medical University of Vienna, Vienna, Austria; ^3^Department of Urology, Okayama University Graduate School of Medicine, Dentistry and Pharmaceutical Sciences, Okayama, Japan; ^4^Department of Urology, Shariati Hospital, Tehran University of Medical Sciences, Tehran, Iran; ^5^Department of Pathology, Medical University of Hamburg, Hamburg, Germany; ^6^Institute for Urology and Reproductive Health, Sechenov University, Moscow, Russia; ^7^Department of Urology, Weill Cornell Medical School, New York, NY, United States; ^8^Department of Urology, University of Texas Southwestern Medical Center, Dallas, TX, United States; ^9^Department of Urology and Andrology, Karl Landsteiner Institute of Urology and Andrology, Vienna, Austria; ^10^Department of Urology, Second Faculty of Medicine, Charles University, Prague, Czechia

**Keywords:** renal cell carcinoma, tyrosine kinase inhibitors, targeted therapy, sarcomatoid histology, therapy regime

## Abstract

**Background:** Sarcomatoid differentiation/histology of renal cell carcinoma (sRCC) in patients with metastatic renal cell carcinoma (mRCC) is still underresearched in current therapy regimes. We aimed to evaluate the impact of sRCC on outcomes in patients with mRCC treated with tyrosine kinase inhibitors (TKIs).

**Methods:** We collected complete data of 262 consecutive mRCC patients from our institutional database for this retrospective study. All patients were treated with TKIs within a single or multimodal treatment approach. All analyses were adjusted for the presence of sRCC. Descriptive statistics as well as uni- and multivariable outcome metrics, including progression-free (PFS) and overall survival (OS) as endpoints were performed.

**Results:** Overall, 18 patients had sRCC (6.9%). Patients with sRCC had more often clear-cell histology (*p* = 0.047), a higher T-stage (*p* = 0.048), and underwent cytoreductive nephrectomy more frequently (*p* < 0.001). The most common first-line TKIs were Sunitinib (65.6%), Sorafenib (19.5%), and Pazopanib (10.3%), respectively. At a median follow-up of 32 months, patients with sRCC had significantly reduced PFS (*p* = 0.02) and OS (*p* = 0.01) compared to patients without sRCC. In multivariable analyses that adjusted for the effects of standard mRCC predictors, the sarcomatoid feature retained its independent association with inferior PFS (HR: 2.39; *p* = 0.007) and OS (HR: 2.37; *p* = 0.001). This association remained statistically significant in subgroup analyses of patients with Sunitinib as first-line therapy (PFS *p* < 0.001; OS: *p* < 0.001).

**Conclusion:** Despite its rare occurrence, our findings confirm sRCC as a powerful predictor for inferior outcomes in mRCC treated with targeted therapies. This suggests a need for more tailored treatment strategies in patients harboring mRCC with sarcomatoid histology to improve oncological outcomes.

## Introduction

Disease management for metastatic renal cell carcinoma (mRCC) has changed since the introduction of tyrosine kinase inhibitors (TKIs). Histological subtypes and variants have shown prognostic value in mRCC (1, 2). However, specific treatment strategies for each histological variation to adapt to the differences in the underlying tumor biology of these phenotypic appearances are not available. Sarcomatoid differentiation/histology of renal cell carcinoma (sRCC) is a histological variant that can occur in the primary tumor, metastases, or both with an incidence of 5–12% in patients with mRCC (3–5). Initially classified as a separate malignancy of the kidney, sRCC is now treated as a histological alteration of carcinomas, that is suggested to replace the initial histological subtype of the malignancy (6). Its pathogenesis is still not fully discovered but chromosomal imbalances and an epithelial-to-mesenchymal transition seem to play a central role in its development and behavior (7, 8).

Sarcomatoid RCC is considered a relevant prognostic factor for unfavorable outcomes in patients with mRCC in the age of primary cytokine treatment as well as in the era of tyrosine kinase inhibitors (TKIs) (3, 5). However, the differential impact of sRCC has not been characterized sufficiently to allow a tailored decision-making pathway for sRCC patients. Therefore, we set out to assess the impact of sRCC on oncological outcomes in mRCC patients in a real-world scenario and to evaluate the differential efficacy of variable treatment algorithms.

We hypothesized that sRCC has a negative impact on survival in mRCC patients in the TKI era and that the currently used therapy regimes, in a real-world setting, are not efficacious enough to provide satisfactory survival outcomes in mRCC patients harboring sRCC.

## Materials and Methods

### Patient Selection

For this retrospective cohort study, we gathered clinical and pathological information of 398 consecutive mRCC patients treated at our tertiary care center between 2005 and 2016. We only included patients with TKI treatment as primary systemic therapy. Patients with prior immunotherapy, metastasectomy only, or missing data were excluded. This left 262 patients for the analyses. Clinical data on the course of the disease as well as baseline characteristics including age, gender, Eastern Cooperative Oncology Group (ECOG) Performance status, Memorial Sloan Kettering Cancer Center (MSKCC) risk score, administered drug therapy and the number of therapy lines, tumor and nodal stage, and data on additional metastasectomy, radiotherapy, and cytoreductive nephrectomy as well as number and locations of metastases were collected and used for analysis.

### Histological Assessment

Surgical specimens were processed according to standard pathological procedures. Tumors were staged and graded according to the American Joint Committee on Cancer–Union Internationale Contre le Cancer TNM classification and the 1998 WHO/International Society of Urologic Pathology consensus classification. Specimens from biopsies were defined as pTx, as the extent of tumor growth could not be specified. Assessment of the primary histology was performed by a dedicated uro-pathologist and classified as clear cell, papillary, or chromophobe RCC as the three predominant subtypes. The presence of sRCC was assessed during a routine pathological workup of the primary or metastasis specimen and defined as the presence of a malignant spindle cell component in the tissue. The proportion of sRCC of the overall specimen in percent was assessed whenever possible.

### Follow-Up

Follow-up was performed according to the current guidelines and patients were seen regularly in our outpatient clinic as previously described (9). Briefly, all patients were seen 4-weekly for a clinical visit and laboratory work-up. Diagnostic imaging of the abdomen and pelvis as well as chest radiography were conducted quarterly. Additional radiographic evaluations (e.g., bone or brain imaging) were performed when clinically indicated. Disease progression was assessed according to the current Response Evaluation Criteria in Solid Tumors (RECIST) version at the time of evaluation (10). Patients who died before recurrence as well as those alive at the end of follow-up were censored for analysis.

### Statistical Analysis

Continuous variables were reported as median, range, and interquartile range (IQR) when non-normal distributed, or as mean and standard deviation when normally distributed. Nominal variables were reported in absolute number and percentage. Baseline patient characteristics were reported and imbalances between patients with and without sRCC were analyzed with a Chi-square test or Fisher's exact-test for nominal and *t*-test for continuous variables. The primary outcome endpoints were progression-free (PFS) and overall survival (OS). We performed survival analyses of global PFS and OS in sRCC compared to non-sRCC patients. Unadjusted Kaplan–Meier estimates were performed to visualize survival outcomes and significant differences in outcomes analyzed with the Log rank test. Uni- and multivariable analyses were performed with a Cox-regression model. Subgroup analyses stratified for the presence of sRCC in different treatment options. First- and second-line therapies were analyzed to evaluate systemic treatment options of sRCC. In the light of the recent debate about cytoreductive nephrectomy, we further analyzed this type of surgical intervention, its association with sRCC, and the resulting impact on survival. Next, we performed subgroup analyses on patients that underwent additional metastasectomy or additional radiotherapy as these treatments are used for symptom control and/or reduction of metastatic burden. Finally, we performed subgroup analyses only in the cohort of patients with sRCC presence, stratifying for the before mentioned treatment options to assess potential effects. Analyses were performed in R 3.4.0 (The R Foundation for Statistical Computing, Vienna, Austria). All results were double-sided and a *p*-value < 0.05 was considered as significant.

## Results

### Baseline Characteristics

**Figure 1:**
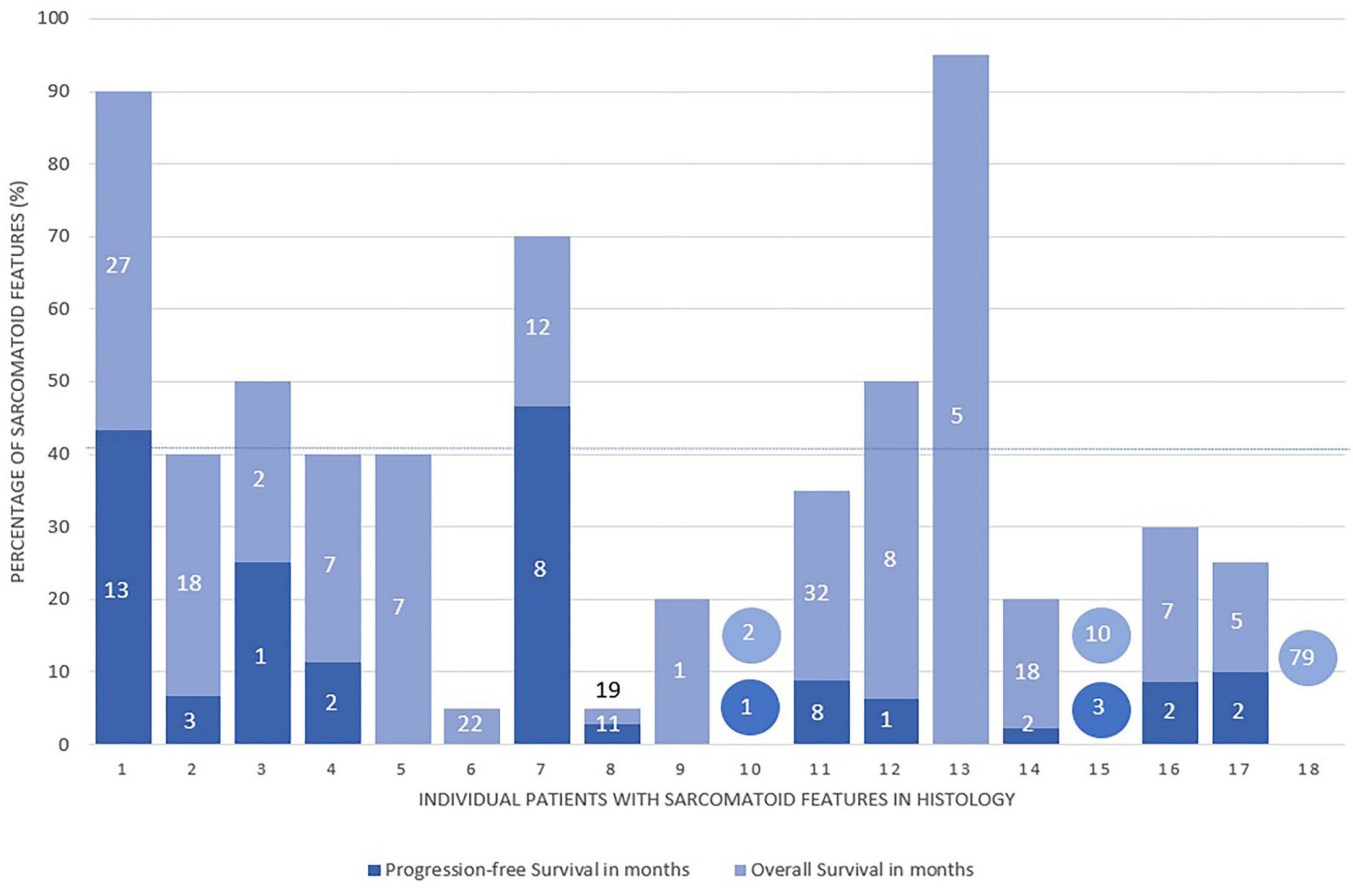
Percentage of sarcomatoid tissue in the histological specimen of 18 patients with sRCC and their according overall survival (OS) and progression-free survival (PFS) in months. Missing values in OS and PFS are censored patients at the end of Follow-up. Values in circles are representing patients with percentage of sarcomatoid features unavailable. Mean percentage of sarcomatoid features in histology (41%; dotted line).

Patient characteristics are presented in Table 1. Overall, 193 patients (73.7%) were men and the median age was 64 years (IQR: 57–71 years). Histology in the entire study cohort were clear cell (*n* = 214; 81.7%), papillary (*n* = 35; 13.4%), and chromophobe RCC (*n* = 7; 2.7%), respectively. Sarcomatoid histology was present in 18 patients (6.9%) and the mean percentage of sarcomatoid features was 41% (5–95%) (Figure 1). In sRCC patients, the underlying RCC histology was clear cell RCC in 15 patients (83.3%), papillary RCC in one patient (5.6%), one patient (5.6%) had complete sarcomatoid differentiation, and one patient (5.6%) mixed histology (clear cell and papillary RCC). Sunitinib was administered as first-line therapy in 172 patients (65.6%), Sorafenib in 51 (24.0%), and Pazopanib in 27 (10.3%), respectively. The most frequent second-line therapies were Everolimus in 53 patients (42.1%), Sunitinib in 30 (23.8%), and Sorafenib in 20 patients (15.9%), respectively. One hundred and four patients (39.6%) underwent cytoreductive nephrectomy, 143 (54.6%) metastasectomy, and 105 underwent additional radiotherapy (40.1%). In general, there was no difference in first- or second-line therapy, or treatment with cytoreductive nephrectomy, metastasectomy, or radiation therapy between patients with or without sRCC histology. In the subgroup of patients with sRCC histology, there was no statistical difference in the type of first-line or second-line therapy, number of treatment lines, metastasectomy, or additional radiotherapy. Patients with sRCC had more often clear cell histology (*p* = 0.047), a higher pT-stage (*p* = 0.017), and underwent cytoreductive nephrectomy more frequently (*p* < 0.001).

**Table 1:** Demographic and clinical characteristics of patients.

**Characteristics**	**Total** **(***N*** = 262)**	**sRCC** **(***N*** = 18)**	**No sRCC** **(***N*** = 244)**	***P*** **-value**
Male sex—*n* (%)	193 (73.7)	14 (77.8)	179 (73.4)	0.79
Median age (IQR)—years	64 (57-71-68)	61 (64–71)	64 (57–72)	0.20
Histology—*n* (%)				**0.047**
Clear cell RCC	214 (81.7)	15 (83.3)	199 (81.6)	
Papillary RCC	35 (13.4)	1 (5.6)	34 (13.9)	
Chromophobe RCC	7 (2.7)	0	7 (2.9)	
Other	6 (2.3)	2 (11.1)	4 (1.6)	
T-stage—*n* (%)				**0.017**
pT1	60 (22.9)	1 (5.6)	59 (24.2)	
pT2	44 (16.8)	2 (11.1)	42 (17.2)	
pT3	110 (42.0)	12 (66.7)	98 (40.2)	
pT4	16 (6.1)	3 (16.7)	13 (5.3)	
pTx^†^	32 (12.2)	0	32 (13.1)	
N-stage—*n*/(%)				0.14
pN0	91 (34.7)	5 (27.8)	86 (35.3)	
pN1	53 (20.2)	7 (38.9)	46 (13.9)	
pNx^†^	118 (45.0)	6 (33.3)	112 (45.9)	
ECOG—*n*/total *n* (%)				0.32
≥2	220/261 (84.3)	17/18 (94.4)	203 /243 (83.5)	
<2	41/261 (15.7)	1/18 (5.6)	40/243 (16.5)	
First-line therapy—*n* (%)				0.95
Sunitinib	172 (65.6)	13 (72.2)	159 (65.2)	
Pazopanib	27 (10.3)	2 (11.1)	25 (10.2)	
Sorafenib	51 (19.5)	3 (16.7)	48 (19.7)	
Other	12 (4.6)	0	12 (4.9)	
Second-line therapy—*n* (%)				0.17
Everolimus	53/126 (42.1)	6/9 (66.7)	47/117 (40.2)	
Sunitinib	30/126 (23.8)	1/9 (11.1)	29/117 (24.8)	
Sorafenib	20/126 (15.9)	0	20/117 (17.1)	
Other	23/126 (18.3)	2/9 (22.2)	21/117 (17.9)	
Number of TKI lines—*n* (%)				0.46
≤2	193 (73.7)	14 (77.8)	179 (73.4)	
>3	69 (26.3)	4 (22.2)	65 (26.6)	
Cytoreductive nephrectomy—*n* (%)	104 (39.6)	15 (83.3)	89 (36.5)	**<0.001**
Karnofsky index—*n*/total *n* (%)				0.40
>80%	173/261 (66.0)	13/18 (72.2)	160/243 (65.8)	
≤80%	88/261 (33.6)	5/18 (27.8)	83/243 (34.2)	
Additional metastasectomy—*n* (%)	143 (54.6)	8/18 (44.4)	135/243 (55.3)	0.26
Additional radiotherapy—*n* (%)	105 (40.1)	8 (44.4)	97 (39.8)	0.70
≥2 Metastatic locations	168 (64.1)	10 (55.6)	158 (64.8)	0.43
**Presence of metastasis in:**
Brain	37 (14.1)	1 (5.6)	36 (14.8)	0.48
Bone	133 (50.8)	9 (50.0)	124 (52.0)	1.00
Lung	193 (73.7)	15 (83.3)	178 (73.0)	0.42
Liver	85 (32.4)	8 (44.4)	77 (31.6)	0.30
Lymph nodes	195 (74.4)	14 (77.8)	181 (74.2)	1.00
Other	149 (56.9)	10 (55.6)	139 (57.0)	1.00
MSKCC score prognosis group—*n* (%)				0.20
Good prognosis	59 (22.5)	2 (11.1)	57 (23.4)	
Intermediate prognosis	153 (58.4)	10 (55.6)	143 (58.6)	
Poor prognosis	50 (19.1)	6 (33.3)	44 (18.0)	

† *Pathological N-stage and/or T-stage was not always assessable in patients without a complete histopathological specimen*.

*ECOG, Eastern Cooperative Oncology Group Performance Score; IQR, interquartile range; MSKCC, The Memorial Sloan Kettering Cancer Center; RCC, Renal cell carcinoma; TKI, Tyrosine kinase inhibitor*.

*Bold values indicate p < 0.05*.

### Overall Survival

**Figure 2:**
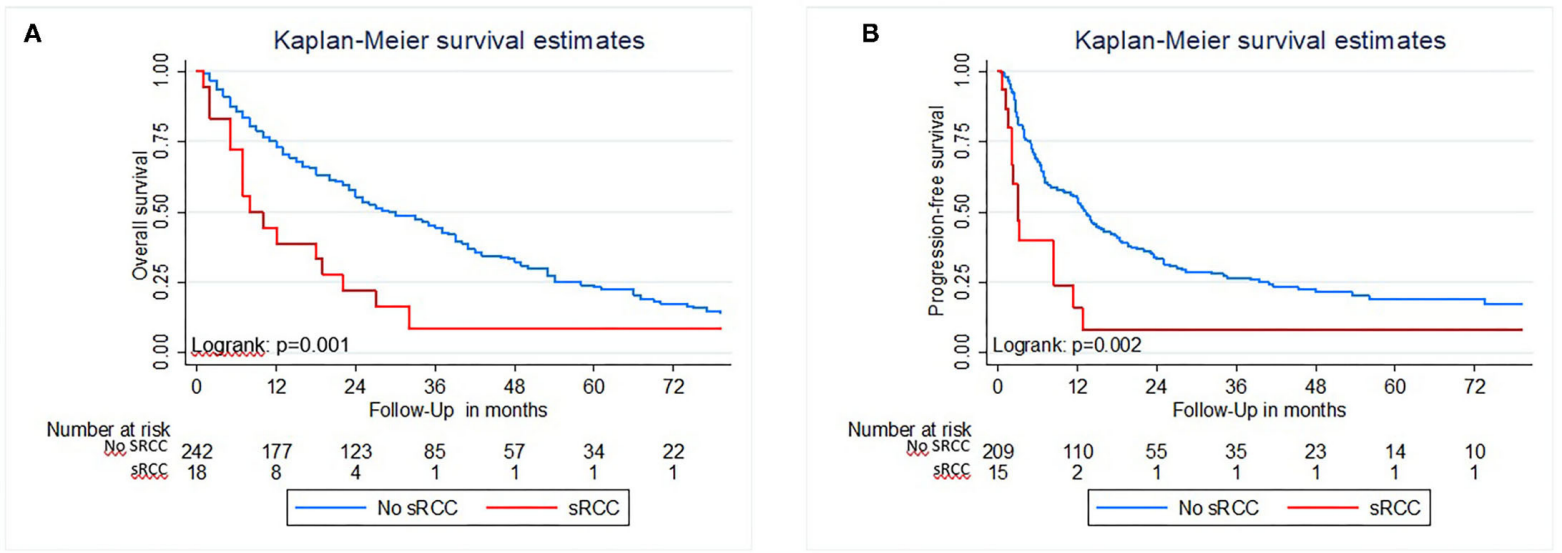
Kaplan Meier estimates of Overall survival **(A)** and Progression-free survival **(B)** of patients with metastatic renal cell carcinoma treated with TKI with and without sarcomatoid features in histology.

Median Follow-Up was 23 months and the median survival of the cohort was 35 months. The presence of sarcomatoid histology was significantly associated with inferior OS in Kaplan–Meier estimation (*p* = 0.001; Figure 2A). In multivariable analysis (Table 2) that adjusted for ECOG performance status, underlying histology, tumor stage and nodal status, cytoreductive nephrectomy, and MSKCC score, respectively, sRCC was an independent predictor for unfavorable OS (HR: 2.37; 95%CI = 1.36–4.11; *p* = 0.001). In addition, higher ECOG Performance status, an undefined pT-stage, presence of nodal metastasis, and an unfavorable MSKCC prognostic score were independent factors for worse OS (all *p* ≤ 0.03). The proportion of sarcomatoid features did not affect the OS in patients with sarcomatoid histology (*p* = 0.3; Figure 1).

**Table 2:** Multivariable analyses of overall and progression-free survival in patients with mRCC treated with TKIs^†^.

	**Overall survival**			**Progression-free survival**		
	**HR**	**95%-CI**	***P*** **-value**	**HR**	**95%-CI**	***P*** **-value**
ECOG PS ≥2 vs. <2	2.01	1.37–2.95	**<0.001**	2.33	1.50–3.62	**<0.001**
Non-ccRCC vs. ccRCC	1.55	1.10–2.20	**0.012**	1.53	1.01–2.32	**0.044**
Sarcomatoid features^‡^	2.37	1.36–4.11	**0.001**	2.39	1.28–4.50	**0.007**
**T-stage**						
T2 vs. T1	1.14	0.70–1.87	0.59	0.92	0.53–1.45	0.69
T3 vs. T1	1.26	0.85–1.85	0.25	1.23	0.67–1.82	0.51
T4 vs. T1	1.68	0.88–3.20	0.11	1.38	0.73–3.11	0.30
Tx vs. T1	1.91	1.11–3.31	**0.02**	1.23	0.66–2.43	0.43
**Lymph node status**						
N1 vs. N0	1.66	1.06–2.59	**0.026**	0.92	0.60–1.40	0.69
Nx vs. N0	1.18	0.82–1.69	0.38	1.06	0.65–1.73	0.81
CN vs. no CN	0.92	0.64–1.33	0.67	1.17	0.77–1.77	0.47
**MSKCC risk score**						
Intermediate vs. good	1.55	1.04–2.31	**0.03**	0.95	0.62–1.64	0.82
Poor vs. good	3.36	2.05–5.52	**<0.001**	1.41	0.81–2.46	0.23
≥3 vs. <3 therapy lines	NS	NS	NS	1.73	1.19–2.51	**0.004**
Liver metastasis present vs. not present	NS	NS	NS	1.10	0.76–1.59	0.60

† *Age, gender, metastasectomy, additional radiotherapy, first- and second-line therapies, as well as locations and number of metastases were omitted from the table as they were not significant in univariable analysis for both endpoints and thus not included in multivariable analysis*.

‡ *Present vs. not present*.

*ccRCC, clear cell renal cell carcinoma; CN, cytoreductive nephrectomy; ECOG PS, Eastern Cooperative Oncology Group Performance status; mRCC, metastatic renal cell carcinoma; MSKCC, Memorial Sloan Kettering Cancer center; NS, not significant in univariable analysis; TKI, tyrosine kinase inhibitor*.

*Bold values indicate p < 0.05*.

### Progression-Free Survival

In Kaplan–Meier analysis, the presence of sRCC was associated with worse PFS (*p* = 0.002; Figure 2B). In a multivariable analysis that adjusted for ECOG performance status, underlying histology, T-stage, nodal status, performed cytoreductive nephrectomy, MSKCC score, and the number of therapy lines, sRCC was an independent predictor for inferior PFS (HR: 2.39; 95%CI = 1.28–4.50; *p* = 0.007). In addition, ECOG performance status, non-clear cell histology, and the number of therapy lines were associated with worse PFS (Table 2).

### Survival Outcomes in Patients With or Without sRCC According to Different Treatment Patterns

Table 3 displays the results of various uni- and multivariable subgroup analyses based on the entire patient population. sRCC was an independent predictor for inferior OS (HR: 2.89; 95%CI = 1.51–5.53; *p* = 0.001) and PFS (HR: 3.94; 95%CI = 1.92–8.09; *p* < 0.001) in multivariable analyses in the subgroup of patients with Sunitinib first-line therapy. In the subgroup of patients undergoing Sorafenib first-line therapy, no multivariable analyses were performed since there was no association between sRCC and outcomes in univariable analyses. In the subgroup of patients with Pazopanib treatment, sRCC was associated with OS and PFS in univariable analysis (both *p* ≤ 0.002), but not an independent predictor in multivariable analyses.

**Table 3:** Uni- and multivariable analyses of association of sRCC and survival endpoints stratified by most common therapy modalities in patients with mRCC treated with TKIs.

	**Overall survival**						**Progression-free survival**					
	**Univariable**			**Multivariable** ^**^‡^**^			**Univariable**			**Multivariable** ^**^‡^**^		
	**HR**	**95%-CI**	***P*** **-value**	**HR**	**95%-CI**	***P*** **-value**	**HR**	**95%-CI**	***P*** **-value**	**HR**	**95%-CI**	***P*** **-value**
**First-line therapy**												
Sunitinib	2.73	1.48–5.03	**0.001**	2.89	1.51–5.53	**0.001**	3.91	1.99–7.70	**<0.001**	3.94	1.92–8.09	**<0.001**
Sorafenib	0.80	0.19–3.31	0.76	NS	NS	NS	0.45	0.06–3.3	0.43	NS	NS	NS
Pazopanib	9.31	1.68–51.74	**0.01**	5.12	0.82–31.84	0.08	10.52	1.45–76.11	**0.02**	5.91	0.77–45.41	0.09
**Second-line therapy**												
Sunitinib	2.39	0.31–18.72	0.41	NS	NS	NS	6.74	0.75–60.29	0.09	NS	NS	NS
Everolimus	3.87	1.56–9.64	**0.004**	7.37	2.51–21.66	**<0.001**	2.62	1.06–6.48	**0.037**	3.66	1.29–10.33	**0.014**
Cytoreductive nephrectomy	1.76	0.97–3.20	0.062	NS	NS	NS	1.55	0.79–3.04	0.20	NS	NS	NS
Metastasectomy	1.33	0.59–3.06	0.49	NS	NS	NS	1.90	0.823–4.37	0.13	NS	NS	NS
Additional radiotherapy^†^	1.45	0.63–3.36	0.38	NS	NS	NS	-	-	-	-	-	-

† *PFS was not analyzed due to radiotherapy as it is mostly performed in patients after progression*.

‡ *Adjusted for variables with significant p-values in univariable analysis [age, gender, ECOG performance status, underlying histology (clear cell vs. non-clear cell histology), cytoreductive nephrectomy, lymph node- and tumor status, metastasectomy, Memorial Sloan Kettering Cancer Center risk group, total number and patterns of therapy lines, and locations and number of metastases]*.

*mRCC, metastatic renal cell carcinoma; NS, not significant in univariable analysis; TKI, tyrosine kinase inhibitor*.

*Bold values indicate p < 0.05*.

In the subgroup analysis of patients with different second-line therapies, sRCC was an independent factor for worse OS (HR: 7.37; 95%CI = 2.51–21.66; *p* < 0.001) and PFS (HR: 3.66; 95%CI = 1.29–10.33; *p* = 0.014) in patients treated with Everolimus. sRCC was not associated with both endpoints in patients treated with Sunitinib in the second-line therapy.

sRCC was not associated with any endpoint in the subgroups of patients that underwent cytoreductive nephrectomy, additional metastasectomy, or additional radiotherapy (all *p* > 0.05), respectively.

### Outcomes of Different Therapeutic Parameters in the Subgroup of sRCC Patients

**Figure 3:**
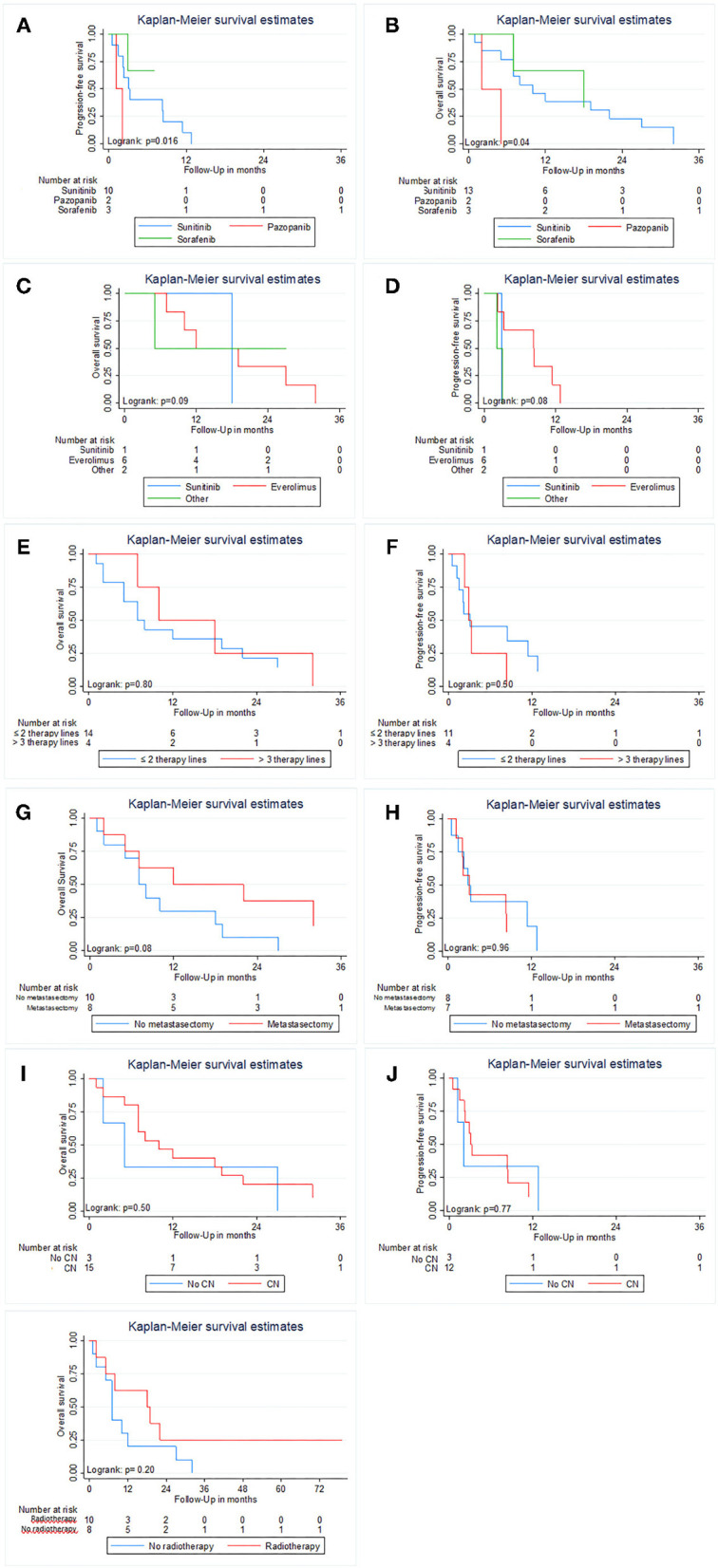
Kaplan Meier estimates of Overall and Progression-free survival in patients with sarcomatoid features in histology stratified by first-line therapy **(A,B)**; MSKCC prognostic risk score **(C,D)**, Gender **(E,F)**, performed metastasectomy **(G,H)**, cytoreductive nephrectomy **(I,J)** and additional radiotherapy **(K)**. Progression-free survival was not analyzed for additional radiotherapy due to its main use after progression of the disease.

Figure 3 displays the results of Kaplan–Meier estimations for overall and PFS in the 18 patients with sRCC. Kaplan–Meier estimates showed a difference in OS and PFS (both *p* ≤ 0.04; Figures 3A,B) between patients treated with Sunitinib, Sorafenib, and Pazopanib. Due to the small patient number, no multivariable Cox regression was performed. There was no difference for each endpoint in any other treatment subgroup analysis (all *p* ≥ 0.05; Figures 3C–K).

## Discussion

We found that sRCC is a strong, independent predictor of inferior OS and PFS in mRCC patients treated with TKIs, despite the rare incidence of this histological variant. Our findings are in congruence with the literature, that supports the role of sRCC as unfavorable outcome prognosticator in mRCC (3, 11–16). Zhang et al. reported worse CSS outcomes when sarcomatoid features were present, with a 6% increase in the risk of cancer-specific mortality for every 10% increase in the proportion of sarcomatoid features (13). However, the impact of the sRCC amount in contrast to its presence is still a point of controversial discussion (14, 15). The mean proportion of sarcomatoid features was 41% in our cohort. A recent meta-analysis of Zhi et al. further confirms the prognostic role of sarcomatoid histology with worse OS, CSS, and PFS in pooled analysis when sarcomatoid histology was present (16). We found a broad range without any clear correlation between the amount and outcomes. Interestingly, variable cut-offs of sRCC amount were reported in the literature to be associated with survival outcomes (11, 12). Due to the small number of patients, in-depth analysis of the impact of sRCC amount on survival was not possible.

Response rates and subsequently outcomes may be associated with the administered systemic therapy (11). We found that sRCC was an independent predictor for inferior survival outcomes in patients treated with Sunitinib. In contrast, we did not find sRCC being an outcome predictor in patients treated with Sorafenib or Pazopanib. This differential effect may be due to group imbalances, variability in sRCC amount, or other factors we could not adjust our analyses for. However, our data are also hypothesis generating, as not all systemic therapies may be equally effective in sRCC patients. When analyzing only patients with sRCC, there was no statistical difference in outcomes between the different first-line therapies, but numbers in this subgroup were very low. Despite representing the same group of drugs, TKIs have inherent differences in their targeting profile (17, 18). In fact, TKIs represented the first-line gold standard therapy in mRCC for almost a decade, but the treatment landscape rapidly expanded in the past few years. A recent *post-hoc* analysis of all the sRCC patients of the Checkmate 214 trial showed a significantly higher overall response rate and a better OS and PFS in intermediate or poor-risk sRCC patients when treated with a checkpoint-inhibitor combination of Nivolumab and Ipilimumab compared to Sunitinib, extending the armamentarium for the treatment of sRCC patients (19). Thus, we need further, large, multi-institutional studies with prospectively collected real-world data that feature the most contemporary landscape.

Literature on second-line therapy in sRCC patients is sparse, and clinical practice usually adheres to the general guideline recommendations. We found that sRCC was an independent factor for worse OS and PFS in patients treated with Everolimus, but not Sunitinib, supporting the role of targeted therapy for sRCC in second-line as well. Despite both drugs being standard of care in the past, also second-line therapy has emerged alongside other contemporary drugs (i.e., Cabozantinib or Nivolumab), that demonstrated survival advantage compared to Everolimus (18, 20). sRCC features differential mutation profiles with a higher mutational burden compared to standard histologies (21, 22). A high mutational burden has been associated with response to targeted therapy (23). A higher PD-L1 expression was an independent predictor for worse survival in patients with mRCC treated with vascular endothelial growth factor (VEGF), as reported by Shin et al. (24). Nevertheless, several biomarkers were analyzed for their impact on survival in both VEGF and MTOR inhibitors by Tantravahi et al., but none showed a significant association (25). Future development of treatment strategies will have to focus on biomarkers and genomic profiles of patients with sRCC to individualize therapy to account for interpatient variability of drug response in any targeted mRCC therapy.

Clinically, the impact of sRCC is of tremendous importance for patients and clinicians alike, as the presence of sRCC is associated with unfavorable tumor biological features (3, 4) and a relevant proportion of >30% in mRCC is attributed to distant spread of the disease (26). We found that sRCC histology was significantly associated with more advanced tumor stages in patients who underwent previous nephrectomy, which is in accordance with the literature (4, 27). In addition, sRCC patients underwent cytoreductive nephrectomy more frequently in our cohort, which is similar to that of a report by Ged et al. (28). Since the CARMENA trial challenged the role of cytoreductive nephrectomy in the TKI era (29), a heterogeneous debate on indications of cytoreductive nephrectomy is ongoing. Shuch et al. reported that patients with sarcomatoid features are less likely admitted for systemic therapy after cytoreductive nephrectomy (27). Nevertheless, according to Alevizakos et al., patients with sRCC had improved CSS when cytoreductive nephrectomy was performed, regardless of tumor stage (30). Further, Ji et al. reported in a recent SEER study that patients, especially those with T1 or T2 sRCC profit in survival outcomes when cytoreductive nephrectomy is performed (31). We did not find any difference in OS or PFS in sRCC patients when cytoreductive nephrectomy was performed; however, the number of patients in the subgroup analyses was very limited which may have masked any association. Due to the more aggressive tumor nature, limited efficacy of systemic TKI therapy and subsequently inferior outcomes in sRCC, intuitively cytoreductive nephrectomy and surgery of metastases seem reasonable in carefully selected patients with a limited metastatic burden. However, these assumptions need to be validated in a larger cohort especially also other systemic treatments including checkpoint inhibition or combination therapies.

Our study is not devoid of inherent limitations due to the retrospective design, potentially introducing an unadjustable selection bias. Due to the low incidence of sRCC, the overall number of sRCC patients in our cohort is small, thus limiting the generalizability of the subgroup analyses results. Unfortunately, the sarcomatoid proportion in the tissue was unavailable for three patients. In addition, objective response to treatment was not present in our retrospective database.

Our findings underscore the rare nature of sRCC as a histological variant in mRCC. sRCC is a strong predictor for unfavorable PFS and OS; however, we observed differences in outcomes according to the administered first- or second-line therapy. These findings support the imperative need for further, contemporary investigations in larger, ideally multicentric, sRCC patient cohorts, for better individually tailored treatment strategies to improve oncological outcomes in a continuously growing treatment landscape.

## Data Availability Statement

The raw data supporting the conclusions of this article will be made available by the authors, without undue reservation.

## Ethics Statement

Ethical review and approval was not required for the study on human participants in accordance with the local legislation and institutional requirements. Written informed consent from the patients was not required to participate in this study in accordance with the national legislation and the institutional requirements.

## Author Contributions

FJ and CK: conceptualization, methodology, formal analysis, writing-original draft, and visualization. CF: data curation and visualization. TK and TH: data curation and investigation. PM: methodology and writing—review and editing. CM: writing—review and editing and data curation. TI and MP: formal analysis. GS and MF: resources and writing—review and editing. SFS: writing—review and editing. RD and MR: supervision, project administration, and writing—review and editing. All authors contributed to the article and approved the submitted version.

## Conflict of Interest

SFS is consulting or advising the following: Astra Zeneca, BMS, Ferring, Ipsen, Jansen, MSD, Olympus, Pfizer, Pierre Fabre, Richard Wolf, Roche, Sanochemia, and Urogen. MR is a speaker for Bayer Healthcare, Bristol Myer Squibb, EUSA Pharma, IPSEN Pharma, Novartis, Roche, and Pfizer. MR is a consultant and/or received honoraria by Bayer Healthcare, Bristol Myer Squibb, IPSEN Pharma, MSD, Novartis, Roche, and Pfizer. The remaining authors declare that the research was conducted in the absence of any commercial or financial relationships that could be construed as a potential conflict of interest. The handling editor declared a shared affiliation with one of the authors, SFS, at the time of review.

## Publisher's Note

All claims expressed in this article are solely those of the authors and do not necessarily represent those of their affiliated organizations, or those of the publisher, the editors and the reviewers. Any product that may be evaluated in this article, or claim that may be made by its manufacturer, is not guaranteed or endorsed by the publisher.

## References

[1] Yip SM Ruiz Morales JM Donskov F Fraccon A Basso U Rini BI. Outcomes of metastatic chromophobe renal cell carcinoma (chrRCC) in the targeted therapy era: results from the international metastatic renal cell cancer database consortium (IMDC). Kidney Cancer. (2017) 1:41–7. 10.3233/KCA-160002 30334003 PMC6179119

[2] Connor Wells J Donskov F Fraccon AP Pasini F Bjarnason GA Beuselinck B. Characterizing the outcomes of metastatic papillary renal cell carcinoma. Cancer Med. (2017) 6:902–9. 10.1002/cam4.1048 28414866 PMC5430092

[3] Kyriakopoulos CE Chittoria N Choueiri TK Kroeger N Lee JL Srinivas S. Outcome of patients with metastatic sarcomatoid renal cell carcinoma: results from the International Metastatic Renal Cell Carcinoma Database Consortium. Clin Genitourin Cancer. (2015) 13:e79–85. 10.1016/j.clgc.2014.08.011 25450036

[4] Naher S Padinharakam S Balakrishnar B Chua W Descallar J Adams D. Patterns of presentation and treatment outcomes of non-clear-cell renal cell carcinoma and sarcomatoid renal cell carcinoma patients in 2 tertiary referral centers in Sydney, Australia. Clin Genitourin Cancer. (2019) 17:e565–e9. 10.1016/j.clgc.2019.02.006 30935815

[5] Cheville JC Lohse CM Zincke H Weaver AL Leibovich BC Frank I. Sarcomatoid renal cell carcinoma: an examination of underlying histologic subtype and an analysis of associations with patient outcome. Am J Surg Pathol. (2004) 28:435–41. 10.1097/00000478-200404000-00002 15087662

[6] Shuch B Amin A Armstrong AJ Eble JN Ficarra V Lopez-Beltran A. Understanding pathologic variants of renal cell carcinoma: distilling therapeutic opportunities from biologic complexity. Eur Urol. (2015) 67:85–97. 10.1016/j.eururo.2014.04.029 24857407

[7] Ito T Pei J Dulaimi E Menges C Abbosh PH Smaldone MC. Genomic copy number alterations in renal cell carcinoma with sarcomatoid features. J Urol. (2016) 195:852–8. 10.1016/j.juro.2015.10.180 26602888 PMC4871784

[8] Jones TD Eble JN Wang M Maclennan GT Jain S Cheng L. Clonal divergence and genetic heterogeneity in clear cell renal cell carcinomas with sarcomatoid transformation. Cancer. (2005) 104:1195–203. 10.1002/cncr.21288 16047350

[9] Janisch F Hillemacher T Fuehner C D'Andrea D Meyer CP Klotzbücher T. The impact of cytoreductive nephrectomy on survival outcomes in patients treated with tyrosine kinase inhibitors for metastatic renal cell carcinoma in a real-world cohort. Urol Oncol. (2020) 38:739.e9–.e15. 10.1016/j.urolonc.2020.04.033 32576526

[10] Therasse P Arbuck SG Eisenhauer EA Wanders J Kaplan RS Rubinstein L. New guidelines to evaluate the response to treatment in solid tumors. European Organization for Research and Treatment of Cancer, National Cancer Institute of the United States, National Cancer Institute of Canada. J Natl Cancer Inst. (2000) 92:205–16. 10.1093/jnci/92.3.205 10655437

[11] Michaelson MD McKay RR Werner L Atkins MB Van Allen EM Olivier KM. Phase 2 trial of sunitinib and gemcitabine in patients with sarcomatoid and/or poor-risk metastatic renal cell carcinoma. Cancer. (2015) 121:3435–43. 10.1002/cncr.29503 26058385

[12] Beuselinck B Lerut E Wolter P Dumez H Berkers J Van Poppel H. Sarcomatoid dedifferentiation in metastatic clear cell renal cell carcinoma and outcome on treatment with anti-vascular endothelial growth factor receptor tyrosine kinase inhibitors: a retrospective analysis. Clin Genitourin Cancer. (2014) 12:e205–14. 10.1016/j.clgc.2014.04.004 24861951

[13] Zhang BY Thompson RH Lohse CM Leibovich BC Boorjian SA Cheville JC. A novel prognostic model for patients with sarcomatoid renal cell carcinoma. BJU Int. (2015) 115:405–11. 10.1111/bju.12781 24730416

[14] Yan Y Liu L Zhou J Li L Li Y Chen M. Clinicopathologic characteristics and prognostic factors of sarcomatoid renal cell carcinoma. J Cancer Res Clin Oncol. (2015) 141:345–52. 10.1007/s00432-014-1740-1 25178995 PMC11824019

[15] Golshayan AR George S Heng DY Elson P Wood LS Mekhail TM. Metastatic sarcomatoid renal cell carcinoma treated with vascular endothelial growth factor-targeted therapy. J Clin Oncol. (2009) 27:235–41. 10.1200/JCO.2008.18.0000 19064974

[16] Zhi H Feng M Liu S Na T Zhang N BiLiGe W. Prognostic significance of sarcomatoid differentiation in patients with metastatic renal cell carcinoma: a systematic review and meta-analysis. Front Oncol. (2020) 10:591001. 10.3389/fonc.2020.591001 33134181 PMC7578539

[17] Choueiri TK Motzer RJ. Systemic therapy for metastatic renal-cell carcinoma. N Engl J Med. (2017) 376:354–66. 10.1056/NEJMra1601333 28121507

[18] Motzer RJ Escudier B McDermott DF George S Hammers HJ Srinivas S. Nivolumab versus everolimus in advanced renal-cell carcinoma. N Engl J Med. (2015) 373:1803–13. 10.1056/NEJMoa1510665 26406148 PMC5719487

[19] McDermott DF Choueiri TK Motzer RJ Aren OR George S Powles T. CheckMate 214 *post-hoc* analyses of nivolumab plus ipilimumab or sunitinib in IMDC intermediate/poor-risk patients with previously untreated advanced renal cell carcinoma with sarcomatoid features. J Clin Oncol. (2019) 37:4513. 10.1200/JCO.2019.37.15_suppl.451331633185

[20] Choueiri TK Escudier B Powles T Tannir NM Mainwaring PN Rini BI. Cabozantinib versus everolimus in advanced renal cell carcinoma (METEOR): final results from a randomised, open-label, phase 3 trial. Lancet Oncol. (2016) 17:917–27. 10.1016/S1470-2045(16)30107-3 27279544

[21] Wang Z Kim TB Peng B Karam J Creighton C Joon A. Sarcomatoid renal cell carcinoma has a distinct molecular pathogenesis, driver mutation profile, and transcriptional landscape. Clin Cancer Res. (2017) 23:6686–96. 10.1158/1078-0432.CCR-17-1057 28710314 PMC5683086

[22] Bi M Zhao S Said JW Merino MJ Adeniran AJ Xie Z. Genomic characterization of sarcomatoid transformation in clear cell renal cell carcinoma. Proc Natl Acad Sci USA. (2016) 113:2170–5. 10.1073/pnas.1525735113 26864202 PMC4776463

[23] Gibney GT Weiner LM Atkins MB. Predictive biomarkers for checkpoint inhibitor-based immunotherapy. Lancet Oncol. (2016) 17:e542–e51. 10.1016/S1470-2045(16)30406-5 27924752 PMC5702534

[24] Shin SJ Jeon YK Cho YM Lee JL Chung DH Park JY. The association between PD-L1 expression and the clinical outcomes to vascular endothelial growth factor-targeted therapy in patients with metastatic clear cell renal cell carcinoma. Oncologist. (2015) 20:1253–60. 10.1634/theoncologist.2015-0151 26424759 PMC4718425

[25] Tantravahi SK Albertson D Agarwal AM Ravulapati S Poole A Patel SB. Survival outcomes and tumor IMP3 expression in patients with sarcomatoid metastatic renal cell carcinoma. J Oncol. (2015) 2015:181926. 10.1155/2015/181926 25688268 PMC4320862

[26] Shuch B Said J LaRochelle JC Zhou Y Li G Klatte T. Histologic evaluation of metastases in renal cell carcinoma with sarcomatoid transformation and its implications for systemic therapy. Cancer. (2010) 116:616–24. 10.1002/cncr.24768 19998348 PMC3162346

[27] Shuch B Said J Rochelle JCL Zhou Y Li G Klatte T. Cytoreductive nephrectomy for kidney cancer with sarcomatoid histology–is up-front resection indicated and, if not, is it avoidable? J Urol. (2009) 182:2164–71. 10.1016/j.juro.2009.07.049 19758641 PMC3175230

[28] Ged Y Chen YB Knezevic A Casuscelli J Redzematovic A DiNatale RG. Metastatic chromophobe renal cell carcinoma: presence or absence of sarcomatoid differentiation determines clinical course and treatment outcomes. Clin Genitourin Cancer. (2019) 17:e678–e88. 10.1016/j.clgc.2019.03.018 31036466 PMC6752712

[29] Mejean A Ravaud A Thezenas S Colas S Beauval JB Bensalah K. Sunitinib Alone or after nephrectomy in metastatic renal-cell carcinoma. N Engl J Med. (2018) 379:417–27. 10.1056/NEJMoa1803675 29860937

[30] Alevizakos M Gaitanidis A Nasioudis D Msaouel P Appleman LJ. Sarcomatoid renal cell carcinoma: population-based study of 879 patients. Clin Genitourin Cancer. (2019) 17:e447–e53. 10.1016/j.clgc.2019.01.005 30799129

[31] Ji B Li D Fu S Zhang Z Yang T Wu Y. A population study to identify candidates for cytoreductive nephrectomy in patients with metastatic sarcomatoid renal cell carcinoma from the surveillance, epidemiology, and end results (SEER) database. Med Sci Monit. (2020) 26:e921297. 10.12659/MSM.921297 32516796 PMC7299061

